# „Risk it“ – warum Frauen ohne Schwangerschaftsabsicht nicht verhüten: Sonderauswertung der BZgA-Studie „frauen leben 3. Familienplanung im Lebenslauf von Frauen“

**DOI:** 10.1007/s00103-021-03439-1

**Published:** 2021-10-14

**Authors:** Cornelia Helfferich, Janet-Lynn Holz, Tilmann Knittel, Laura Olejniczak, Franziska Schmidt

**Affiliations:** Sozialwissenschaftliches Forschungsinstitut zu Geschlechterfragen an der Evangelischen Hochschule (EH) Freiburg, SoFFI F./FIVE, Bugginger Str. 38, 79114 Freiburg, Deutschland

**Keywords:** Unbeabsichtigte Schwangerschaften, Ungewollte Schwangerschaften, Verhütung, Kinderwunsch, Familienplanung, Unintended pregnancies, Unwanted pergnancies, Contraception, Desire for children, Family planning

## Abstract

**Hintergrund:**

Trotz allgemein bekannten Verhütungsmethoden und flächendeckendem Zugang zu Verhütungsmitteln lässt sich beobachten, dass Frauen in westlichen Industriegesellschaften auch bei fehlender Schwangerschaftsabsicht oftmals nicht verhüten und schwanger werden.

**Ziel der Arbeit:**

Die in diesem Beitrag durchgeführten Analysen zielen darauf ab, die Verbreitung des Phänomens der Nichtanwendung von Verhütung für Deutschland einzuschätzen und ein besseres Verständnis für die Gründe zu gewinnen, warum Frauen eine unbeabsichtigte Schwangerschaft riskieren.

**Material und Methoden:**

Anhand von quantitativen Befragungsdaten zu 17.400 Schwangerschaften und von 116 offenen qualitativen Interviews aus der im Auftrag der Bundeszentrale für gesundheitliche Aufklärung (BZgA) durchgeführten Studie „frauen leben 3. Familienplanung im Lebenslauf von Frauen“ sind Aussagen zur Verbreitung der Nichtverwendung von Verhütungsmethoden trotz fehlender Schwangerschaftsabsicht und zu den dahinterliegenden Gründen möglich.

**Ergebnisse:**

Die angegebenen Gründe für die Nichtverwendung von Verhütung lassen sich 3 sehr unterschiedlichen Motivlagen zuordnen: a) *Spielen mit einem Kinderwunsch*, was von knapp einem Drittel genannt wird, b) *individuelle und strukturelle Hürden*, wie z. B. gesundheitliche Vorbehalte oder zu hohe Kosten, und c) *irrtümliche Annahme, nicht schwanger werden zu können*. Es zeigt sich, dass diese Motive je nach biografischer Situation unterschiedlich verbreitet sind.

**Diskussion:**

Die Ergebnisse weisen auf die Notwendigkeit eines differenzierteren Verständnisses unbeabsichtigter Schwangerschaften und zwar sowohl in der Forschung als auch der Prävention hin.

Als Limitation und damit als Bedarf für künftige Forschungen erweist sich, dass im Rahmen der Studie der Einfluss des (Sexual‑)Partners auf das Verhütungsverhalten der Frauen und auf die Gewolltheit der Schwangerschaft anhand der erhobenen Daten nicht untersucht werden kann.

## Abstract (en)

**Background:**

Despite widespread access to contraception, it is observed that women without pregnancy intention do not use contraception and become pregnant.

**Aim of this paper:**

The analyses conducted in this paper aim to assess the prevalence of the phenomenon of non-use of contraception for Germany and to gain a better understanding of the reasons why women risk unintended pregnancy.

**Materials and methods:**

Using quantitative survey data on 17,400 pregnancies and 116 open qualitative interviews from the study “Women’s lives 3: Family planning in women’s lives” conducted on behalf of the Federal Centre for Health Education (BZgA), statements can be made on the prevalence of non-use of contraceptive methods despite a lack of pregnancy intention and on the underlying reasons.

**Results:**

The reasons given for not using contraception can be assigned to three very different motives: a) playing with a desire to have children, which is mentioned by just under one third; b) individual and structural barriers, such as health reservations or too high costs; and c) not using contraception because of the erroneous assumption of not being able to get pregnant. It can be seen that these motives vary in prevalence depending on the biographical situation.

**Discussion:**

The results point to the need for a more differentiated understanding of unintended pregnancies in both research and prevention. A limitation and thus a need for future research is that the influence of the (sexual) partner on the contraceptive behaviour of women and on the intentionality of the pregnancy cannot be examined within the framework of the study.

## Hintergrund

In den meisten der hoch industrialisierten westlichen Gesellschaften ist zu beobachten, dass Frauen ohne Kinderwunsch verhüten könnten, dies zu einem Teil aber nicht tun und damit eine unbeabsichtigte Schwangerschaft riskieren. Einerseits ist der Zugang zu effektiver Verhütung weitgehend möglich und breit akzeptiert^1^ [2, 6], andererseits tritt ein nicht unbeträchtlicher, über die Zeit weitgehend stabiler Anteil der Schwangerschaften – nämlich zwischen einem Drittel und gut der Hälfte – ungewollt oder zumindest unbeabsichtigt ein [7–9].

Der Zugang zu Verhütungsmitteln ist ein Kernaspekt der reproduktiven und sexuellen Gesundheit [10, 11], deren Bedeutung als Menschenrecht von dem im Juni 2021 vom EU-Parlament angenommenen Matić-Bericht betont wird [12, 13]. Die Verfügbarkeit der nötigen Informationen, Kenntnisse und Mittel zur Verhütung bildet die Voraussetzung sowohl für die Realisierung des Rechts, Sexualität angenehm, sicher, frei von Zwang, Diskriminierung und frei von Angst vor einer (sozial stigmatisierten, „illegitimen“ [14]^2^) ungewollten Schwangerschaft zu leben, als auch für die Realisierung des Rechts auf selbstbestimmte Familienplanung [11, 12, 14]. Zudem wird der barrierefreie und breite Zugang zu Verhütungsmitteln als wirksame Strategie zur Senkung der Verbreitung unbeabsichtigter Schwangerschaften betrachtet [7, 17].

Dagegen verweisen empirische Forschungen auf eine eingeschränkte Anwendung von Verhütungsmethoden – trotz grundsätzlicher Verfügbarkeit und bestehenden Bedarfs. Gemäß den Ergebnissen von Befragungsstudien zum Verhütungsverhalten aus den Vereinigten Staaten berichten zwischen 11 % und 46 % der Frauen, die zum Befragungszeitpunkt nicht beabsichtigten schwanger zu werden, dass sie nicht oder nur inkonsistent verhüten [18–20]. Für Frankreich liegen Ergebnisse vor, dass bei etwa jeder dritten ungeplanten Schwangerschaft nicht verhütet wird [9].

In der internationalen Forschung wird das Phänomen der Nichtanwendung von Verhütungsmethoden im Wesentlichen unter zwei Perspektiven diskutiert. Der erste Diskussionsstrang konzentriert sich auf (alltagspraktische) individuelle und strukturelle Hürden bei der Nutzung von Verhütungsmethoden [21]. Es zeigt sich hierbei, dass die Gründe, nicht zu verhüten, von der Verhütungsmethode, den eigenen Einstellungen und Einschätzungen wie auch den Einflüssen des (Sexual‑)Partners abhängen. Gründe, Verhütungsmittel nicht anzuwenden, können in den Nebenwirkungen dieser Mittel, ihren Kosten oder technischen Schwierigkeiten in der Anwendung liegen [22–25]. Als weitere Motive für die Nichtverwendung von Verhütungsmitteln wird die Unerwartetheit des Geschlechtsverkehrs und die Überzeugung angeführt, nicht fruchtbar zu sein bzw. ein geringes Schwangerschaftsrisiko zu haben [19, 22, 23]. Auch Einstellungen und Werthaltungen können zu einer Ablehnung der Verwendung von Verhütungsmitteln führen [19]. Ebenfalls wird der Einfluss des sozialen Umfeldes diskutiert, so führen Einstellungen Dritter – etwa aus dem Familien- und Freundeskreis – sowie die Ablehnung der Verwendung von Verhütungsmitteln des (Sexual‑)Partners zu nicht verhütetem Geschlechtsverkehr [19, 22].

Ein zweiter Strang der Forschungsdiskussion befasst sich mit dem Konzept der Schwangerschaftsintention selbst. Statt als Gegensatzpaar von gewollten und ungewollten Schwangerschaften wird die Schwangerschaftsintention in aller Regel als Kontinuum bzw. mehrdimensionales Konstrukt – und als möglicherweise sogar widersprüchliche Gemengelage von „Wollen“ und „Ablehnen“ einer Schwangerschaft – betrachtet. Den Standard in der retrospektiven Abfrage von Schwangerschaftsintentionen stellen die im Rahmen des US-amerikanischen *National Surveys of Family Growth (NSFG)* entwickelten Differenzierungen dar. Dort wurde, neben einer ersten affektiven Dimension *Desire* (dt. Verlangen, Wunsch), die differenziert wurde in „wanted“, „unwanted“ (dt. gewollt, ungewollt), eine zweite Dimension des *Timing* eingeführt, die auf die Gewolltheit des Zeitpunkts der Schwangerschaft abzielt. Hierbei wird unterschieden, ob Schwangerschaften auf den Zeitpunkt hin gewollt („at the right time“), früher als eigentlich gewollt („mistimed“) oder ungewollt („unwanted“) eingetreten sind [26]. Schwangerschaften, die ungewollt oder zum falschen Zeitpunkt eingetreten sind, werden als unbeabsichtigte („unintended“) Schwangerschaften zusammengefasst.

Im Zusammenhang mit der Schwangerschaftsintention wird zudem eine nicht bzw. inkonsistent verwendete Verhütung als Indiz für eine ambivalente Haltung gegenüber einer Schwangerschaft diskutiert [19, 27–30]. Die empirischen Untersuchungen von Crosby et al. [28] und Frost et al. [31] aus den Vereinigten Staaten belegen zum Teil auf spezifische Zielgruppen bezogen entsprechende Zusammenhänge. In einer weiteren US-amerikanischen Studie [19] berichten Frauen, bei denen die Schwangerschaft zum falschen Zeitpunkt („mistimed“) eingetreten ist, im Vergleich zu Frauen mit einer ungewollten Schwangerschaft zu einem – wenn auch nicht statistisch signifikanten – höheren Anteil von einem inkonsistenten Verhütungsverhalten vor der Schwangerschaft. Die Nutzung von Verhütung wird zudem vielfach auch bei empirischen Erhebungen zur „(Un) Geplantheit von Schwangerschaften“ berücksichtigt (ein Konzept, das allerdings von dem der „Schwangerschaftsintention“ zu unterscheiden ist), so auch im Rahmen des etablierten *London Measure of Unplanned Pregnancy*. Innerhalb einer 6 Dimensionen umfassenden Skala wird die Nichtverwendung von Verhütungsmitteln als ein Indiz für Geplantheit gewertet [18]. Dahinter steckt die Annahme, dass sich die Nutzung von Verhütungsmitteln als Ausdruck der Stärke des Wunsches, eine Schwangerschaft zu vermeiden, lesen lässt.

Für Deutschland gibt es bisher nur wenige Studien, die diese international diskutierten Forschungsstränge aufgreifen. Zu nennen ist hier die repräsentative Studie der Bundeszentrale für gesundheitliche Aufklärung (BZgA) zum Verhütungsverhalten Erwachsener in Deutschland. Die Ergebnisse zeigen, dass die befragten Erwachsenen auf die Verwendung von Verhütungsmitteln verzichten, weil ein Kinderwunsch besteht, eine Schwangerschaft vorliegt, aufgrund von Infertilität bzw. der Menopause oder aufgrund fehlender sexueller Aktivität [5]. Allerdings wurde bei dieser Befragung nicht dezidiert die Schwangerschaftsintention erhoben. Damit fehlen bisher aussagekräftige Daten in Deutschland zu dem Zusammenhang zwischen fehlender Verwendung von Verhütungsmethoden und unbeabsichtigten Schwangerschaften.

Entsprechende Aussagen zur Situation in Deutschland sollen im Folgenden auf Grundlage einer Auswertung der Daten der Mixed-Methods-Studie „frauen leben 3“ abgeleitet werden. Aufbauend auf einer überblickhaften Vorstellung des Studiendesigns und der Messkonzepte wird zunächst auf die Verbreitung unbeabsichtigter – mit oder ohne Verhütung eingetretener – Schwangerschaften eingegangen. Daran anschließend werden auf Grundlage der quantitativen Daten als auch der qualitativen Interviews die Motive für die Nichtanwendung von Verhütungsmethoden untersucht, um diese abschließend zu diskutieren, in den Forschungsstand einzuordnen und ihre Relevanz für Forschung und Präventionspraxis darzulegen.

## Methode

Die vorgestellten Ergebnisse basieren auf Daten der Studie „frauen leben 3“, die im Auftrag der BZgA vom Sozialwissenschaftlichen Forschungsinstitut zu Geschlechterfragen Freiburg (SoFFI F.) durchgeführt wird. Der Fokus der Studie „frauen leben 3“ liegt auf Familienplanung und Sexualität im Lebenslauf der retrospektiv befragten Frauen. Dazu gehören Bereiche wie Partnerschaften, Verhütungsverhalten, Schwangerschaften und Schwangerschaftsabbrüche. Zwischen 2011 und 2018 erfolgte in 3 Erhebungsphasen in 12 deutschen Bundesländern eine repräsentative standardisierte Telefonbefragung von 14.500 Frauen der Geburtsjahrgänge 1967 bis 1997 mit insgesamt rund 17.400 zwischen 1983 und 2018 ausgetragenen oder abgebrochenen Schwangerschaften.^3^ Die Feldorganisation und -arbeit wurde vom Befragungsinstitut KANTAR Deutschland (vormals TNS Emnid) durchgeführt.

Die Stichprobenziehung erfolgte auf Grundlage des Master-Samples der Arbeitsgemeinschaft deutscher Marktforschungsinstitute (ADM) für generierte Telefonnummern. Auswahlgesamtheit für dieses Sample waren damit die Privathaushalte in den ausgewählten Regionen der Bundesrepublik Deutschland mit mindestens einem Festnetzanschluss. Um auch Haushalte zu erreichen, die nicht in Verzeichnissen aufgeführt werden, wurden per „Random-Last-Two-Digits-RL(2)D-Verfahren“ in Anlehnung an das sogenannte Gabler-Häder-Verfahren der Universität Mannheim zusätzlich neue Nummern generiert. Aus diesem Telefonnummernpool wurde die eigentliche Stichprobe per Zufallsauswahl gezogen.

Die Befragung wurde zusätzlich als Screening genutzt, um mit 116 Frauen, die nach spezifischen biografischen Merkmalen ausgewählt wurden, offene qualitative Interviews – u. a. zu unbeabsichtigten Schwangerschaften – zu führen. Die folgenden Auswertungen beziehen sich auf die Daten aus allen 3 Erhebungsphasen.

Die Operationalisierung der Schwangerschaftsintention – also der Gewolltheit oder Ungewolltheit einer Schwangerschaft – erfolgte bei „frauen leben 3“ in enger Anlehnung an das oben dargestellte Konzept des *National Survey of Family Growth *[26]. Neben den Antwortmöglichkeiten von „gewollt und auch der Zeitpunkt war richtig“/„die Schwangerschaft hätte früher eintreten sollen“ („wanted“), „ungewollt“ („unwanted“), „gewollt, aber später*“ *(„mistimed“) wurde ebenfalls die Antwortkategorie „ich war unentschieden, zwiespältig“ aufgenommen. Die 3 zuletzt genannten Antwortmöglichkeiten bilden gemeinsam die breitere Kategorie „unbeabsichtigt“. Es wurden sowohl ausgetragene als auch abgebrochene Schwangerschaften in die Analysen miteinbezogen. Schwangerschaftsabbrüche werden der Kategorie „ungewollt“ zugeordnet. Über die Schwangerschaftsintention im engeren Sinne hinaus wurde zudem die emotionale Reaktion auf die Schwangerschaft erfragt, welche auch bei unbeabsichtigten Schwangerschaften häufig durchaus positiv ausfällt [32–34].

Das Verhütungsverhalten der Befragten bei unbeabsichtigten Schwangerschaften wurde anhand der Frage: „Wenn Sie sich zurückerinnern: Sind Sie damals unter Verhütung schwanger geworden?“, erfasst. Bei Verneinung dieser Frage wurde ohne die Vorgabe von Antwortmöglichkeiten nach den Gründen für das Absetzen bzw. die Nichtverwendung der Verhütungsmittel befragt. Die Antworten wurden von den spezifisch geschulten Interviewerinnen Kategorien zugeordnet.

## Ergebnisse

### Prävalenz unbeabsichtigter Schwangerschaften mit und ohne Verhütung

**Figure 1:**
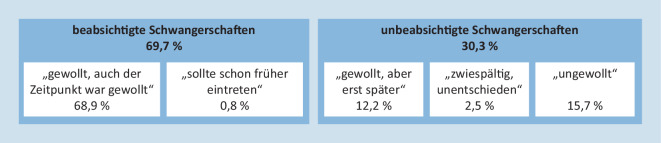

Die Auswertung der standardisierten Daten der Studie „frauen leben 3“ zeigt, dass etwa 30 % aller dokumentierten Schwangerschaften aus Sicht der befragten Frauen unbeabsichtigt eingetreten sind (Abb. 1). Unter diesen unbeabsichtigten Schwangerschaften befinden sich 15,7 % dezidiert ungewollte Schwangerschaften. 12,2 % aller Schwangerschaften waren gewollt, aber erst zu einem späteren Zeitpunkt und bei 2,5 % aller Schwangerschaften wurde die Intention als unsicher bzw. zwiespältig bezeichnet.

Bei der deutlichen Mehrzahl (61,8 %) der unbeabsichtigten Schwangerschaften wurde nicht verhütet. Bezogen auf alle Schwangerschaften sind damit 18,1 % aller Schwangerschaften aufgrund von unterlassener Verhütung trotz fehlender klarer Schwangerschaftsabsicht eingetreten.

### Alter bei Eintritt unbeabsichtigter Schwangerschaften

Im Vergleich der Altersgruppen sind unbeabsichtigte Schwangerschaften am meisten bei Frauen im Alter zwischen 25 und 30 Jahren verbreitet (Tab. 1)^4^. Hochgerechnet auf 1000 Frauen dieser Altersgruppe treten 142,3 unbeabsichtigte Schwangerschaften ein. Bei unter 20-jährigen und über 40-jährigen Frauen sind unbeabsichtigte Schwangerschaften deutlich seltener (40,9 bzw. 31,2 unbeabsichtigte Schwangerschaften je 1000 Frauen). Ungewollte Schwangerschaften sind damit vor allem in den Altersgruppen verbreitet, in denen auch gewollte Schwangerschaften am häufigsten eintreten.

**Table 1:** 

Altersgruppe	Unbeabsichtigte Schwangerschaften pro 1000 Frauen			Gewollte Schwangerschaften pro 1000 Frauen
	Gesamt	Ohne Verhütung eingetreten	Unter Verhütung eingetreten	
Unter 20 Jahre	40,9	20,2	19,3	9,3
20 bis unter 25 Jahre	106,6	58,0	45,4	117,5
25 bis unter 30 Jahre	142,3	91,6	46,3	378,9
30 bis unter 35 Jahre	116,0	74,9	37,7	492,3
35 bis unter 40 Jahre	78,2	50,8	22,9	261,6
40 bis unter 45 Jahre	31,2	14,5	15,1	74,3

Sonderauswertung frauen leben 3

Frauen der Geburtsjahrgänge 1967 bis 1997, *n* = 14.500 Befragte

Hinweis: Aufgrund von teilweise fehlenden Angaben zur Verhütung liegt die Summe der ausgewiesenen Häufigkeiten der unbeabsichtigten Schwangerschaften ohne und unter Verhütung zum Teil geringfügig niedriger als die ausgewiesene Häufigkeit unbeabsichtigter Schwangerschaften gesamt

In den mittleren Altersgruppen wird bei ungewollten Schwangerschaften überdurchschnittlich häufig nicht verhütet. Treten unbeabsichtigte Schwangerschaften bei Frauen unter 20 Jahre und über 40 Jahre etwa zu gleichen Anteilen mit und ohne Verhütung ein, wird bei unbeabsichtigten Schwangerschaften von Frauen zwischen 25 und 40 Jahren doppelt so häufig die Verhütung weggelassen wie verhütet wird. Mithin wird bei unbeabsichtigten Schwangerschaften genau in den Altersgruppen seltener verhütet, in denen auch gewollte Schwangerschaften besonders häufig vorzufinden sind. Diese Altersspanne von 25 Jahren bis 35 Jahre entspricht der gesellschaftlich anerkannten Altersnorm als der *richtige Zeitpunkt *der Familiengründung [15].

Wie weitere lebenssituationsbezogene Auswertungen der „frauen leben 3“-Daten zeigen, treten mit einem Anteil von 91,2 % die große Mehrheit der unbeabsichtigten Schwangerschaften bei Frauen ein, die sich in einer festen Paarbeziehung befinden. Unbeabsichtigte Schwangerschaften außerhalb fester Partnerschaften bilden somit die Ausnahme. Bei ohne Verhütung eingetretenen unbeabsichtigten Schwangerschaften liegt der Anteil der Frauen in Paarbeziehung mit 92,0 % vergleichbar hoch.

Bei 60,6 % aller unbeabsichtigten Schwangerschaften (unabhängig davon, ob diese unter oder ohne Verhütung eingetreten sind) war die Befragte zuvor kinderlos, bei knapp einem Viertel (23,9 %) hatte sie ein Kind und bei 15,6 % 2 oder mehr Kinder. Nach Parität differenziert zeigt sich, dass bei unbeabsichtigten Schwangerschaften am häufigsten nicht verhütet wurde, wenn die Mutter zuvor ein Kind geboren hatte (69,9 %). War die Frau zuvor kinderlos, wurde in 60,7 % der unbeabsichtigten Schwangerschaften nicht verhütet, hatte sie bereits 2 oder mehr Kinder, war dies bei lediglich 53,6 % der Fall.

## Gründe für die Nichtanwendung von Verhütung

Bei unbeabsichtigten, ohne Verhütung eingetretenen Schwangerschaften lassen sich anhand der in der Befragung angegebenen Gründe für die Nichtanwendung von Verhütung 3 wesentliche Motivlagen (Tab. 2) erkennen, auf die in den folgenden Abschnitten im Detail eingegangen wird. Die in Tab. 2 unter „Sonstiges“ aufgeführten Gründe lassen sich keiner Motivlage zuordnen. Da bei dieser Frage in der Befragung lediglich Spontannennungen ohne Antwortvorgaben erhoben wurden, liegen für weitere 19,6 % der Befragten keine bzw. keine inhaltlich interpretierbaren Antworten vor.

**Table 2:** 

Genannte Gründe für die Nichtanwendung von Verhütung nach Motivlagen	Anteil der unbeabsichtigten Schwangerschaften, die ohne Verhütung eingetreten sind (%)
*1. Keine strikte Schwangerschaftsablehnung*	
Spielen mit Kinderwunsch, Leichtsinn	30,8
*2. Individuelle und strukturelle Hürden*	
Gesundheit, Nebenwirkungen	15,6
Ablehnung von Verhütung, keine Methode passt, Verhütungsmüdigkeit	5,6
Kosten, zu teuer	3,4
Gründe für Absetzen Verhütung bzw. für Nichtverhütung: Gründe, die beim Partner liegen	2,3
Gerade beim Wechsel der Methode gewesen, noch keine wirksame neue Methode	2,2
Hürden bei Beschaffung und Zugang: Aufwand (z. B. Rezept, fehlende Info, Ausland, Chaos)	1,7
*3. Irrtümliche Annahme, nicht schwanger werden zu können*	
Dachte, ich kann nicht schwanger werden, wegen Alter oder Diagnose bei sich oder Partner	7,8
*Sonstiges*	
Einmaliges Vergessen, Versehen, sonst wurde verhütet	8,2
Kein Partner, kein Sex, nicht mit Sex gerechnet	2,1

Sonderauswertung der Studie „frauen leben 3“

*n* = 3135 unbeabsichtigte, ohne Verhütung eingetretene Schwangerschaften

Als Prozentwerte sind die relativen Häufigkeiten der Nennungen einzelner Gründe bezogen auf unbeabsichtigte Schwangerschaften ohne Verhütung ausgewiesen. Mehrfachnennungen waren möglich

### Motivlage 1: Keine strikte Schwangerschaftsablehnung

Der am häufigsten genannte Grund ist* Spielen mit dem Kinderwunsch, Leichtsinn* mit einer Nennung bei 30,8 % (Tab. 2) aller unbeabsichtigten, ohne Anwendung von Verhütung entstandenen Schwangerschaften. Dies kann als Hinweis gewertet werden, dass eine weggelassene oder inkonsequente Verhütung in der Tendenz auf eine „etwas mehr gewollte“ respektive weniger strikt abgelehnte Schwangerschaft hindeutet. Gestützt wird diese Interpretation dadurch, dass *Spielen mit dem Kinderwunsch, Leichtsinn* besonders häufig bei grundsätzlich (aber erst später) gewollten Schwangerschaften (36,0 %) und bei einer zwiespältigen Haltung (33,7 %) als Grund für das Weglassen von Verhütung genannt wird – bei dezidiert ungewollten Schwangerschaften dagegen nur von 24,1 %. Zudem wird das Spielen mit dem Kinderwunsch überdurchschnittlich häufig von Frauen in für gewollte Schwangerschaften typischen Altersgruppen und nach dem ersten Kind als Grund genannt.

Schließlich zeigt sich auch ein Zusammenhang zwischen (Nicht‑)Verhütung und der Reaktion auf unbeabsichtigte Schwangerschaften. Zwar wurden unbeabsichtigte Schwangerschaften, die ohne Verhütung eingetreten sind, nahezu gleich häufig abgebrochen (20,9 %) wie unter Verhütung eingetretene Schwangerschaften (22,9 %). Bei den letztlich ausgetragenen unbeabsichtigten Schwangerschaften war die Erstreaktion auf nicht unter Verhütung eingetretene Schwangerschaften allerdings deutlich positiver als bei Schwangerschaften trotz Verhütung: Bei nichtverhüteten Schwangerschaften reagierten 74,2 % der Befragten auf die Feststellung der Schwangerschaft (eher) erfreut, bei unter Verhütung eingetretenen Schwangerschaften lediglich 53,3 %.

Der Rückgriff auf die qualitativen Interviews ermöglicht im Rahmen von „frauen leben 3“ eine nähere Analyse der subjektiven Sicht der Frauen. Hier finden sich Beispiele, bei denen die Verhütung aus einer Situation heraus weggelassen wurde, in der über den Kinderwunsch nicht eindeutig und klar zu entscheiden war.

Zum einen betraf dies Frauen, bei denen zu wenig gegen ein Kind sprach, um das Thema „abzuhaken“, aber auch zu wenig für ein Kind, um eine gewollte Schwangerschaft zielgerichtet anzustreben. Eine Schwangerschaft konnte daher nicht als gewollte, sondern nur als nicht beabsichtigte eintreten. Diese Schwierigkeiten treffen systematisch auf Frauen zu, die eine biografisch gewachsene Ablehnung von Kindern in ihrem Leben abmindern und in fortschreitendem Alter umschwenken und sich vorstellen können, Mutter zu werden. Im Zuge des Wandels der Lebensplanung entstehen so strukturell bedingte Phasen der Ambivalenz hinsichtlich einer Schwangerschaft.

Zum anderen betraf dies Frauen, bei denen bei einem grundsätzlichen Kinderwunsch das Umfeld oder die Bedingungen gegen ein Kind sprechen („das Herz sagt Ja, der Kopf sagt Nein“). Die qualitativen Interviews zeigen soziale Diskriminierungen „unvernünftiger Geburten“, mit denen Frauen aus Sicht ihres Umfelds etwa ihre beruflichen Chancen verspielen.

### Motivlage 2: Individuelle und strukturelle Hürden

In der „frauen leben 3“-Befragung zeigen sich ebenfalls die in der Forschungsliteratur dokumentierten praktischen Hürden bei der Verhütung (Tab. 2). Am häufigsten (15,6 %,) werden *Gesundheit, Nebenwirkungen* als Gründe für den Verzicht auf Verhütung genannt, gefolgt von *Ablehnung von Verhütung, keine passende Methode, Verhütungsmüdigkeit *mit 5,6 % sowie *Kosten, zu teuer *mit 3,4 % als weitere praktische Hürden.

Ein zu hoher Aufwand, fehlende Rezepte oder fehlende Informationen als Hürden bei Beschaffung von bzw. Zugang zu Verhütung wurden mit einem Anteil von 1,7 % insgesamt selten genannt und betreffen – wie tiefergehende Analysen des Datensatzes zeigen – vor allem junge Frauen unter 20 Jahre.

### Motivlage 3: Irrtümliche Annahme, nicht schwanger werden zu können

Bei 7,8 % (Tab. 2) der unbeabsichtigten, ohne Anwendung von Verhütungsmethoden eingetretenen Schwangerschaften wurde auf Verhütung in der irrtümlichen Annahme verzichtet, mit der Begründung: *dachte, ich kann nicht schwanger werden, wegen Alter oder Diagnose bei sich oder Partner*. Besonders häufig wird dies von Frauen, die bei Eintritt der unbeabsichtigten Schwangerschaft 35 Jahre oder älter waren, als Grund für die Nichtverhütung genannt (14,2 %), bei jungen Frauen unter 20 Jahre dagegen nur selten (3,9 %).

## Diskussion

Die ausgeführten Analysen der Daten der Studie „frauen leben 3“ belegen, dass das Phänomen der Nichtanwendung von Verhütung trotz fehlender Schwangerschaftsabsicht auch in Deutschland verbreitet ist. Bei 61,8 % der unbeabsichtigten Schwangerschaften wurde keine Verhütungsmethode angewendet. Damit liegt der Anteil für Deutschland höher als jener, der in der Studie von Bajos et al. [9] für Frankreich ermittelt wurde. Eine nähere Auseinandersetzung mit dem Widerspruch zwischen Nichtanwendung von Verhütung und fehlender Schwangerschaftsabsicht ist sowohl für die wissenschaftliche Auseinandersetzung mit dem theoretischen Konzept und der Messung der Schwangerschaftsintention als auch für die Konzeption wirksamer gesundheitspolitischer Strategien zur Prävention unbeabsichtigter Schwangerschaften angezeigt.

Der Blick auf die genannten Gründe für die Nichtanwendung von Verhütung trotz fehlender Schwangerschaftsabsicht – soweit er im Rahmen der Studie „frauen leben 3“ möglich ist – offenbart eine erhebliche Heterogenität der dahinterstehenden Motivlagen. So lassen sich für ungefähr ein Drittel der Schwangerschaften *individuelle und strukturelle Hürden* als Gründe ausmachen, darunter der am häufigsten genannte Grund *Gesundheit, Nebenwirkungen. *Hier könnte eine gewisse Skepsis oder kritische Haltung gegenüber Verhütungsmitteln eine Rolle spielen, die sich auch in den Ergebnissen einer Studie der BZgA [5] widerspiegelt. Dort wurde aktuell ein Rückgang der Verwendung der Antibabypille sowie eine kritische Haltung gegenüber hormonellen Verhütungsmethoden aufgezeigt.

Die Kosten der Verhütung sind dagegen in der Gesamtbetrachtung als nachrangiger Grund für die Nichtanwendung von Verhütung zu sehen, auch wenn nicht vernachlässigt werden darf, dass – wie in einer anderen Sonderauswertung des „frauen leben 3“-Datensatzes [35] gezeigt werden konnte – bei Frauen in besonders prekären wirtschaftlichen Situationen der zeitweilige Verzicht auf Antibabypille oder Spirale aus Kostengründen verbreitet ist.

Die Verbreitung der – bei etwa einem Drittel der ohne Verhütung eingetretenen unbeabsichtigten Schwangerschaften genannten – Motivlage *Spielen mit dem Kinderwunsch, Leichtsinn *verweist zusammen mit den Ergebnissen der qualitativen Interviews darauf, dass die Schwangerschaftsintention notwendigerweise als mehrdimensionales Konstrukt konzipiert werden muss. In Übereinstimmung mit internationalen Studien [28, 31, 36] legen die qualitativen Ergebnisse nahe, dass die Nichtverwendung von Verhütungsmitteln bei nicht vorhandener Schwangerschaftsabsicht bei einem Teil der Frauen als eine weniger strikte Ablehnung einer Schwangerschaft interpretiert werden kann.

Festzuhalten ist zudem, dass in der Lebenswirklichkeit der Frauen vielfach nur bedingt Klarheit hinsichtlich der Schwangerschaftsabsicht besteht. Für die Prävention weisen die Ergebnisse darauf hin, dass über die Vermittlung von Informationen, die Stärkung der Verhütungskompetenz und einen niedrigschwelligen Zugang zu Verhütung hinaus sowohl Frauen als auch Männer in der Praxis dabei unterstützt werden sollten, Klarheit und Sicherheit über ihren eigenen Kinderwunsch zu gewinnen.

Die Diskussion der Ergebnisse verweist unmittelbar auf einige Limitationen der Studie. Die Erfassung der Gründe für die Nichtanwendung von Verhütung erfolgte zu rudimentär für tiefergehende Analysen. Auch könnten gezielte Erhebungen zur Häufigkeit der (Nicht‑)Verwendung von Verhütungsmitteln sowie der subjektiven Einschätzung der Effektivität verwendeter Methoden noch genauere Einblicke in die komplexe Motivlage der Frauen ermöglichen.

Als weitere designbedingte Einschränkung der Studie ist zu nennen, dass ausschließlich Frauen selbst befragt wurden. In der Forschungsliteratur wird dagegen auch der Einfluss des (Sexual‑)Partners auf das Verhütungsverhalten der Frauen und letztendlich auch auf die Gewolltheit der Schwangerschaft betont [37–39]. Higgins et al. [27] konnten zeigen, dass die Nichtverwendung von Verhütungsmethoden im Zusammenhang mit einer ambivalenten Haltung zum Eintritt einer Schwangerschaft häufiger bei den befragten Männern als bei den Frauen zu beobachten ist. Daraus ergibt sich, dass der Einbezug der (Sexual‑)Partner in zukünftige Forschung unabdingbar dafür ist, um Erkenntnisse über das komplexe Phänomen der Nichtverwendung von Verhütungsmitteln bei fehlender Schwangerschaftsintention zu gewinnen [27, 40].

## Footnotes

1 Einen historischen Überblick für den europäischen Raum zu der Verwendung von Verhütungsmitteln und ihrer gesellschaftlichen Akzeptanz gibt Busch [1]. In dem Bericht des Department of Economic and Social Affairs der United Nations zeigt sich weltweit die Zunahme der Anwendung von Verhütungsmethoden seit 1970. Erwartet wird, dass sich dieser Trend bis 2030 fortsetzt [2]. Laut Bericht der BZgA für Deutschland liegt der Prozentsatz der Anwendung von Verhütungsmitteln bei sexuell aktiven Erwachsenen in den letzten Jahren bei ca. 70 % [3–5].

2 Die Trennung von sexuellem Akt und Reproduktion beendete die Disziplinierung weiblicher Sexualität im Sinne einer Restriktion auf einen *legitimen* Kontext – die Ehe nämlich –, in der Nachwuchs explizit erwartet wurde [14–16].

3 Bis Ende 2020 wurden in einer vierten Erhebungsphase in den restlichen 4 Bundesländern Bayern, Hessen, dem Saarland und Sachsen-Anhalt standardisierte Telefoninterviews mit 4500 Frauen sowie weitere 20 qualitative Gespräche geführt. Aufgrund der laufenden Datenaufbereitung konnten diese Befragungsdaten noch nicht berücksichtigt werden.

4 Die Häufigkeit unbeabsichtigter Schwangerschaften ist ausgewiesen als Quotient von berichteten Schwangerschaften eines Altersjahres je 1000 Frauen, zu denen für das entsprechende Altersjahr Informationen vorliegen. Das Vorgehen ist mit der üblichen Berechnung der zusammengefassten Geburtenziffern (Total Fertility Rate) nach Altersgruppen vergleichbar.
